# Neuropsychiatric symptoms and diagnosis of grey matter heterotopia: A case-based reflection

**DOI:** 10.4102/sajpsychiatry.v23i0.923

**Published:** 2017-03-28

**Authors:** Gian Lippi

**Affiliations:** 1 Department of Psychiatry, Faculty of Health Sciences, University of Pretoria, South Africa; 2 Forensic Unit, Weskoppies Hospital, South Africa

## Abstract

Neuropsychiatric symptoms can be related to less common underlying neuropsychiatric conditions – in this case report, the condition discussed is that of grey matter heterotopia (GMH). The patient presented with a history of prominent aggression, impulsivity and manipulative and attention-seeking behaviour. Episodes of depression and incidents of deliberate self-harm and suicide attempts had been reported. Neuropsychiatric symptoms included anxiety, a labile mood, delusional thinking and auditory hallucinations. Testing revealed some cognitive difficulties and severe impairment of frontal lobe functions. A magnetic resonance imaging (MRI) scan of his brain revealed the presence of GMH, which had previously been misdiagnosed as tuberous sclerosis. An MRI scan of the brain is the special investigation of choice for the correct diagnosis of GMH. The pathognomonic finding is that of heterotopic grey matter abnormally located within areas of white matter. Defective foetal neuronal migration between the third and fifth month of pregnancy can lead to GMH, which can present later on in childhood or adolescence with epilepsy, intellectual impairment or reading difficulties. During the late teenage years or early adulthood, a wide variety of neuropsychiatric symptoms may be present, which can lead to diagnostic difficulties.

## Introduction

A 39-year-old, unemployed Caucasian male was arrested for assault with intent to cause grievous bodily harm involving an incident of extreme violence which occurred in a psychiatric ward where he was being treated as an involuntary mental health care user under the *Mental Health Care Act* No. 17 of 2002.^1^ Because of his history of treatment for mental illness, he was sent for psychiatric observation in terms of Sections 77, 78 and 79 of the *Criminal Procedures Act* No. 51 of 1977, as amended.^2^ The observation was done at Weskoppies Hospital, a tertiary psychiatric hospital in Pretoria, South Africa. During this period of evaluation, he was diagnosed with an interesting underlying condition of grey matter heterotopia (GMH), which had previously been misdiagnosed as tuberous sclerosis (TS).

## Presenting concerns

During the 13 years preceding the observation, he had regularly been engaged with psychiatric services and had presented with multiple, wide-ranging psychiatric symptoms. Most prominent was his impulsivity and difficulty in controlling his aggression, which had led to multiple documented incidents of physical aggression. He had also regularly displayed manipulative and attention-seeking behaviour. He had been suffering from anxiety and a labile mood. He presented with episodes of depression, and there were incidents of deliberate self-harm and suicide attempts. Delusional thinking and perceptual disturbances in the form of auditory hallucinations were less prominent. Because of the complexity of his clinical presentation, he had been diagnosed with a number of different psychiatric disorders including borderline personality disorder; antisocial personality disorder; major depressive disorder; bipolar disorder; schizophrenia and schizoaffective disorder, bipolar type.

## Background history

The patient had been a breech presentation during pregnancy and developed hydrocephalus secondary to an occipital encephalocoele. Normal flow of cerebrospinal fluid was restored with the resection of the encephalocoele. Early developmental milestones were normal. He completed primary schooling in a mainstream school but presented with learning difficulties. He was bullied and teased by other learners and had no friends. His aggression started at a young age with destruction of property and assaults of fellow learners being reported. Because of his poor academic performance, he attended a special high school where he completed his schooling.

There was also a history of alcohol and cannabis abuse:

- At age 5, he was diagnosed with epilepsy, which continued into adulthood and resulted in him being diagnosed with psychotic disorder due to epilepsy at a later stage.
- The patient’s biological father also has epilepsy and a cousin on the paternal side of the family suffers from intellectual disability.
- The patient had had a computerised tomography (CT) scan of his brain 11 years ago, and was subsequently misdiagnosed with TS. A psychiatric diagnosis of personality change due to TS was also subsequently made.

## Clinical findings

During the observation period, demanding and attention-seeking behaviour was observed, but there were no episodes of physical aggression. He displayed a distinct lack of remorse for the assault for which he had been charged, and he held the victim responsible for what had transpired. His mood was labile, but he was not depressed. He was objectively angry at times and admitted to feeling anxious. Subtle persecutory and grandiose delusions were identified, and he admitted to experiencing thoughts of reference.

Some cognitive impairment was apparent. He scored 25 out of 30 on the Folstein Mini Mental State Examination, and specific sifting revealed severe impairment of frontal lobe functions. Attention, judgement, planning and response inhibition were very poor. He also presented with impairment in constructional, sequencing and set-shifting abilities. Intelligence quotient (IQ) testing revealed a score within the range of borderline intellectual functioning.

A neurological examination revealed no motor, sensory, cranial nerve or cerebellar abnormalities.

A physical examination during the observation period revealed none of the characteristic dermatological lesions associated with TS, so the diagnosis was queried (also because specific genetic testing for TS had not been done).

## Diagnostic focus and assessment

**FIGURE 1:**
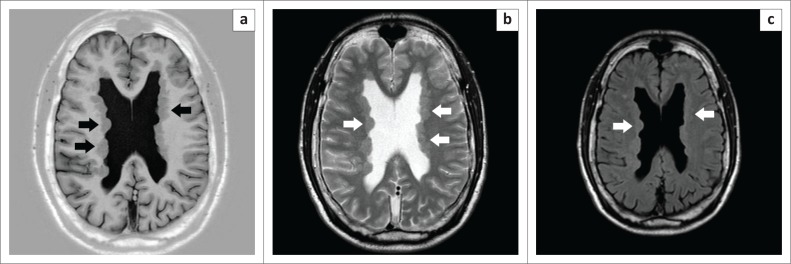
MRI scan sections of the patient’s brain indicating the presence of GMH. (a) A T1W IR SENSE axial section of the brain with arrows indicating bilateral and asymmetrical PNH with heterotopic grey matter stretching all along the ventricular walls, even present anterior to the anterior horns. Note that the shade of grey is the same as that of the cortical grey matter (the same signal intensity), which confirms that it is grey matter – the pathognomonic finding in GMH. (b) The arrows point towards the PNH where the typical nodular appearance of the heterotopic grey matter can be seen on this T2W TSE SENSE axial section of the brain. (c) On this T2W CLEAR SENSE GAD enhanced axial section of the brain, one can see how the subependymal nodules cause bulging of the ventricular walls, as pointed out by the arrows. GMH – grey matter heterotopia; T1W – Tau 1 weighted; T2W – Tau 2 weighted; FLAIR – fluid-attenuated inversion recovery; IR – inversion recovery; FFE – fast field echo; SENSE – sensitivity encoding; TSE – turbo spin echo; CLEAR – constant level appearance; GAD – gadolinium; PNH – periventricular nodular heterotopia.

A decision was made to refer him for a magnetic resonance imaging (MRI) scan of his brain, and the findings proved to be invaluable in making the correct diagnosis; we were fortunate that this form of imaging was available, as there is a limited availability of MRI facilities outside of the major cities in South Africa. The scan revealed the presence of bilateral, diffuse subependymal nodules along the walls of the lateral ventricles, which had the same signal intensity as grey matter on T1W, T2W, FLAIR and IR sequences, with no blooming artefact on T2W FFE and no enhancement post contrast (Figure 1). The radiologists concluded that the findings are more in keeping with a diagnosis of GMH and that with TS one would have expected calcification and enhancement of subependymal nodules.

## Follow-up, therapeutic focus and outcomes

Following the findings and reports related to his observation, he was referred to Weskoppies Hospital as a State Patient in terms of Section 42 of the *Mental Health Care Act* No. 17 of 2002.^1^ He continues to receive care, treatment and rehabilitation at this institution. The emphasis of this case report is the diagnosis of GMH and its related neuropsychiatric symptoms rather than therapeutic interventions, outcomes and prognosis; therefore, further information about these areas of patient management is not provided.

## Ethical considerations

Permission to report on this case was granted by the Research Ethics Committee of the Faculty of Health Sciences of the University of Pretoria. The patient signed informed consent for the case report to be published after having been assessed to have had the capacity to do so.

## Discussion

### Embryology and pathology

GMH is a neuronal migration disorder characterised by the presence of normal grey matter neurons and glial cells, which are abnormally located in clusters within areas of white matter.

During embryonic development of the brain, neuroblasts migrate away from the germinal layer of the ventricular neuroepithelium, mostly along the tracks of radial glial fibres.^3,4^ In cases of GMH, the neuronal migration is prematurely halted resulting in the formation of heterotopic nests in the white matter.^4,5,6,7^ There is differing opinion as to whether GMH can also form due to neurons losing their way during migration, migrating in the wrong direction or migrating too far (into the subpial space), and as to whether these heterotopic nests establish abnormal interconnections and exert abnormal trophic influences on surrounding tissue or whether only a limited number of axons exit these nests resulting in limited connectivity with other areas of the brain.^5,6^

GMH can be classified as being laminar or nodular. Both types have been described as occurring in the same patient.^4^ Laminar heterotopia consists of bands of neurons between the cortex and lateral ventricles; it is therefore also known as band heterotopia or double cortex. Nodular heterotopia consists of round nodules of normal neurons and glial cells with no laminar organisation and can be classified according to the location of these nodules as: (1) subcortical and (2) subependymal (periventricular) – hence the term periventricular nodular heterotopia (PNH).^3,5,6^ Because of the location of PNH, close to the subependymal germinal matrix, the nodules often cause bulging of the ventricular walls.^3^

Five different groups of PNH have been identified: (1) bilateral and symmetrical; (2) bilateral single-noduled; (3) bilateral and asymmetrical; (4) unilateral and (5) unilateral with extension to neocortex.^3^

### Epidemiology

GMH is more common than was once thought. Heterotopic tissue cannot always be seen on a CT scan,^8^ so it is since the regular use of MRI scans – which can more accurately discriminate between grey and white matter^4^ – that GMH is being reported much more frequently than had been expected,^6,8^ both as an incidental finding and in symptomatic patients.^6^ GMH may constitute about 15% of cortical developmental malformations and may be found in about 2% of patients with epilepsy.^8^ In a study of 16 patients who were being evaluated for intractable epilepsy, all of them were found to have GMH – both laminar and nodular forms were discovered.^9^ The prevalence of GMH in the general population is not known.^8^ There are conflicting reports as to whether the phenomenon has been found in normal persons.^5,7,10^ In another study investigating the prevalence of GMH using MRI scans for diagnostic purposes, none of the 75 normal persons, who made up the control group of the study, had GMH.^10^

### Genetics

Specific genetic mutations have been identified in patients with certain types of PNH, and indeed other forms of GMH, some of which even have differing heritance patterns.^3,6,11^ It has been discovered that genetics play a major role in the development of bilateral PNH. Bilateral and symmetrical cases, in particular, are characterised by a familial occurrence and a positive family history of epilepsy, even though it is associated with a clear female predominance.^3^ Bilateral and symmetrical PNH is an X-linked-dominant disease with linkage mapping to chromosome *Xq28*.^3,6,8,11,12^ Mutations of the filamin A (FLNA) or filamin I (FLNI) gene have been identified.^3,8,11,12^

The development of GMH is thought to be due to a combination of this underlying genetic susceptibility coupled with an environmental insult caused by, for example, toxins of viral infections during the time of foetal brain development in the form of neuronal migration between the third and fifth month of pregnancy.^2,5,7,10^

### Grey matter heterotopia and psychiatric disorders

Preliminary findings indicate that GMH is more common in patients with schizophrenia with a prevalence rate of 1.8% being found in a study where none of the normal individuals in the control group were found to have the disorder.^7,10^ Even though this prevalence is low, there is a possibility that it is actually higher because defective neuronal migration may not be detectable with current imaging equipment.^10^ Microscopic evidence that may support this theory has been reported in the form of defectively migrated, abnormally located nicotinamide-adenine dinucleotide phosphate-diaphorase neurons being found in the white matter of the frontal and temporal lobes of patients with schizophrenia.^5,7,9^ A different study reported that patients with schizophrenia were found to have heterotopic neurons in the CA1 areas of the hippocampus. Abnormal hippocampal and temporal lobe activity is frequently associated with epilepsy and psychotic disorders.^5^ Theories of schizophrenia being a neurodevelopmental disorder are gathering support. GMH may be a gross or macroscopic manifestation of microscopic abnormalities found in a sub-population of patients with schizophrenia.^7^

The two disorders have a similar proposed aetiopathogenesis of an underlying genetic susceptibility coupled with an environmental insult.^7,10^ Both disorders also have a high prevalence of agenesis of the corpus callosum and are associated with a finding of ventricular dilatation.^7^ Even though the proposed aetiology of GMH is very similar to that of schizophrenia, it is clearly not specific to the disorder.^10^ GMH has been discovered in a number of psychiatric disorders, which are listed in Box 1.

BOX 1 Psychiatric disorders where grey matter heterotopia has been discovered.^3,4,5,7,8,10,11,12,13^

- Intellectual disability
- Schizophrenia
- Unspecified schizophrenia spectrum disorder
- Schizoaffective disorders
- Major depressive disorder
- Bipolar disorder
- Delirium
- Language disorder
- Autism spectrum disorder
- Attention-deficit hyperactivity disorder
- Somatic symptom disorder

The most common neuropsychiatric clinical picture is that of intellectual disability, which ranges from mild to severe in nature, even though many patients with GMH present with normal intellectual functioning.^4,11^

### Grey matter heterotopia and other medical conditions

Certain types of GMH have also been found to be associated with metabolic disorders such as neonatal adrenoleukodystrophy, connective tissue disorders such as Ehlers-Danlos syndrome and a number of central nervous system malformations such as Chiari II malformations, agenesis of the corpus callosum, encephalocoeles and myelomeningocoeles.^3,4,6^

### Presenting symptoms

The clinical picture is determined by the type of GMH and the location of the heterotopic tissue. Most of the presenting symptoms are common to all types of GMH but may differ in severity among the different types. Certain symptoms have been described only in certain types of GMH. For the purposes of this overview of the subject, presenting symptoms of GMH are discussed as a whole.

The most common initial symptoms are those of epileptic seizures.^3,4,5,6,9,11^ Absence seizures are the most common.^6^ Epilepsy usually develops during the second decade of life.^3,6^ Developmental delay is common but neurological deficits are usually only seen in more severe cases of GMH with mild motor, sensory and visual defects being reported.^3,6,9^ Dyspraxia and problems with fine motor skills have also been found.^12^ Neurological deficits and developmental delay, in particular, depend on the type and severity of the GMH as well as the location of the lesions. Patients with severe unilateral subcortical GMH often present with hemiplegia, yet it is not uncommon for a person with GMH to present without neurological deficits or developmental delay.^6,10^

### Neuropsychiatric symptoms

An overview of the literature reveals that most publications have concentrated on the neurological presentations (especially epilepsy), radiological findings and the genetics of GMH. Neuropsychiatric symptoms are less well researched and possibly underrecognised.^12^ Box 2 provides a list of these neuropsychiatric symptoms. The development of all these symptoms can be independent of epilepsy because they were found to be present before the onset of seizures in many children with GMH.^11^

BOX 2 Neuropsychiatric symptoms of grey matter heterotopia.^3,4,5,7,8,11,12,15^

**Symptoms commonly presenting during childhood**

- Dyslexia and other learning difficulties
- Deficits in:
  - Reading fluency
  - Spatial orientation
  - Planning
  - Constructional abilities
  - Attention
  - Processing speed
  - Adaptive skills
  - Other areas of executive functioning
- Problems in areas of:
  - Social skills
  - Leadership
  - Functional communication
- Hyperactivity
- Rage
- Aggression
- Violence
- Lack of remorse for problematic behaviour

**Mood symptoms**

- Mood lability
- Depressive symptoms
  - Low mood
  - Low self-esteem
  - Insomnia and nightmares
  - Social withdrawal
  - Hopelessness
  - Deliberate self-harm
  - Suicidal ideation and suicide attempts
- Manic symptoms
  - Elevated mood
  - Racing thoughts
  - Decreased need for sleep
  - Distractibility
  - Irritability
  - Agitation
  - Impulsivity
  - Sexual inhibition and sexually inappropriate behaviour
  - Financial and social indiscretions

**Psychotic symptoms**

- Thought form disorder
- Auditory and visual hallucinations
- Paranoid ideation
- Referential thinking
- Vague delusions (more so than systematised delusions)
- Inappropriate affect

**Other, less common symptoms**

- Anxiety
- Panic attacks
- Obsessive-compulsive symptoms
- Phobias
- Disorientation and ‘confusion’

### Case-related discussion

The findings on the MRI scan of the patient in the case report are consistent with that of GMH. The bilateral, diffuse subependymal nodules along the walls of the lateral ventricles suggest a diagnosis of PNH.^3,5,6^ The fact that the signal intensity of the nodules on the various sequences referred to in the case report is the same as grey matter is a pathognomonic diagnostic finding on the MRI.^7,9,10^ With TS, the subependymal nodules are usually isointense to hypointense compared with white matter.^7,8,10^ Other imaging findings suggestive of TS such as calcifications, cortical tubers and white matter hamartomas were also absent in this case.^4,14^

An absence of the typical dermatological signs of TS – hypomelanotic macules or ash leaf spots (found in up to 90% of patients with TS), confetti lesions, poliosis, facial angiofibromas or adenoma sebaceum, shagreen patches and ungual fibromas^14^ – alerted the clinician to the possibility that the patient did not suffer from TS. Because of these clinical and radiological findings, the previous diagnosis of TS was considered to be incorrect, and he was subsequently diagnosed with bilateral and asymmetrical PNH.

Examining his background history and the clinical findings again, retrospectively, after the new diagnosis of GMH was made, reveals interesting findings, some of which are unique to this case, and others that are consistent with what has been described in the literature.

This patient with PNH being born with an encephalocoele is noteworthy since a concurrent development of the two anomalies has been described.^6^ Even with such a potentially debilitating birth defect, his developmental milestones were reportedly normal, which is not uncommon in patients with GMH. The absence of neurological deficits other than those which are more neuropsychiatric in nature is also not an unusual finding.^6,10^

His developing epilepsy at age 5 is earlier than the usual second decade of life reported in the literature for this patient population.^3,6^ With his father also suffering from epilepsy and a cousin on the paternal side of the family having intellectual disability, there are two family members with the two most common features of GMH.

The patient has presented with a large number and a varying spectrum of neuropsychiatric symptoms over the years. Probably, the most prominent features of his presentation are aggression, impulsive violence, inability to control anger and lack of remorse for his violent acts. All of these symptoms have been described in patients with GMH.^3,8,11,12,15^ With such a combination of symptoms present from childhood, it is not surprising that he had previously been diagnosed with antisocial personality disorder and personality change due to TS.

‘Mood swings’ have been described in patients with GMH.^12^ In this case, the patient’s labile mood could partly explain previous diagnoses of bipolar disorder and schizoaffective disorder, bipolar type.

His episodes of depression and his history of previous deliberate self-harm and suicide attempts, all of which have been reported in relation to GMH,^8,12^ make his previous diagnoses of major depressive disorder and borderline personality disorder understandable.

His prominent anxiety, even in the absence of mood abnormalities, is noteworthy and can be present in patients with the same condition.^3,12^

Intellectual disability of varying severity (including that which is mild in nature), specific learning disorder and executive dysfunction have been associated with GMH.^3,5,11,12,13^ This patient had a history of learning difficulties and attended a special school during his high school years. He presented with executive dysfunction and impairment in other areas of frontal lobe functioning. Taking into account his history of cognitive impairment since childhood, his level of adaptive functioning and the results of his IQ test, he was subsequently diagnosed with borderline intellectual functioning.

His vague, subtle, mild paranoia and referential thinking are similar to what has been described in other patients with GMH.^8,12^ His auditory hallucinations were also a far less prominent feature than many of the other neuropsychiatric symptoms he had experienced. When one also takes into account both this pattern of psychotic features and his history of symptoms of depression and mood lability, his variety of previous diagnoses that primarily present with psychotic symptoms are comprehensible.

From a diagnostic point of view, there was never a clear period of him having experienced auditory hallucinations during a period in which he was not abusing cannabis and he was also not experiencing these hallucinations during the observation period and had not abused substances in the months leading up to the observation. With him presenting only with non-bizarre delusions, his symptoms did not meet the criteria for a diagnosis of schizophrenia. A case has been described where such a patient with GMH was diagnosed with, in Diagnostic and Statistical Manual of Mental Disorders, fifth edition (DSM-5) terminology,^16^ an unspecified schizophrenia spectrum disorder,^8,16^ which is what the patient in the case report was eventually diagnosed with. However, with him having developed epilepsy at such a young age, an argument can easily be made that the diagnosis should be psychotic disorder due to epilepsy, if the opinion is that the psychotic symptoms are secondary to the epilepsy. If his symptoms had met the diagnostic criteria for schizophrenia, it could be argued that that is what the diagnosis should be and that the GMH is an incidental finding due to the low incidence of GMH in schizophrenia. It can also be concluded that since the psychotic symptoms are not typical of schizophrenia, they may be secondary to the GMH, in which case the diagnosis would be psychotic disorder due to bilateral and asymmetrical periventricular nodular grey matter heterotopia.

## Conclusion

GMH is a neuronal migration disorder which is more common than was once thought in the time before MRI scans were regularly used. The condition most frequently presents with epilepsy but intellectual disability of varying severity is also commonly found. GMH has not only been associated with schizophrenia, but also with a wide range of neuropsychiatric symptoms, many of which may be present in the same patient. When that is the case, diagnosis may be difficult.

This case highlights that when patients present with many different neuropsychiatric symptoms, a possible diagnosis of GMH should be considered and that they should be referred for an MRI scan, where possible, to look for the pathognomonic findings of heterotopic grey matter abnormally located within areas of white matter.
